# Predictive modelling of deliberate self-harm and suicide attempts in young people accessing primary care: a machine learning analysis of a longitudinal study

**DOI:** 10.1007/s00127-022-02415-7

**Published:** 2023-02-28

**Authors:** Catherine M. McHugh, Nicholas Ho, Frank Iorfino, Jacob J. Crouse, Alissa Nichles, Natalia Zmicerevska, Elizabeth Scott, Nick Glozier, Ian B. Hickie

**Affiliations:** 1 Brain and Mind Centre, University of Sydney, 94 Mallett Street, Camperdown, Sydney, NSW 2042 Australia; 2 St Vincent’s Hospital, Sydney, Australia; 3 School of Medicine, University of Notre Dame Australia, Sydney, Australia; 4 School of Psychiatry, University of Sydney, Sydney, Australia; 5 Discipline of Psychiatry, University of New South Wales, Sydney, Australia

**Keywords:** Suicidal behaviour, Suicide attempt, Deliberate self-harm, Machine learning, Youth, Adolescence

## Abstract

**Purpose:**

Machine learning (ML) has shown promise in modelling future self-harm but is yet to be applied to key questions facing clinical services. In a cohort of young people accessing primary mental health care, this study aimed to establish (1) the performance of models predicting deliberate self-harm (DSH) compared to suicide attempt (SA), (2) the performance of models predicting new-onset or repeat behaviour, and (3) the relative importance of factors predicting these outcomes.

**Methods:**

802 young people aged 12–25 years attending primary mental health services had detailed social and clinical assessments at baseline and 509 completed 12-month follow-up. Four ML algorithms, as well as logistic regression, were applied to build four distinct models.

**Results:**

The mean performance of models predicting SA (AUC: 0.82) performed better than the models predicting DSH (AUC: 0.72), with mean positive predictive values (PPV) approximately twice that of the prevalence (SA prevalence 14%, PPV: 0.32, DSH prevalence 22%, PPV: 0.40). All ML models outperformed standard logistic regression. The most frequently selected variable in both models was a history of DSH via cutting.

**Conclusion:**

History of DSH and clinical symptoms of common mental disorders, rather than social and demographic factors, were the most important variables in modelling future behaviour. The performance of models predicting outcomes in key sub-cohorts, those with new-onset or repetition of DSH or SA during follow-up, was poor. These findings may indicate that the performance of models of future DSH or SA may depend on knowledge of the individual’s recent history of either behaviour.

**Supplementary Information:**

The online version contains supplementary material available at 10.1007/s00127-022-02415-7.

## Introduction

Self-harm and suicidal behaviours are common in young people and are associated with significant morbidity and increased risk of repeat suicidal behaviour and premature death by suicide [1, 2]. Population studies of young people who engage in self-harm have suggested that the majority of self-harm resolves without intervention [1]. Yet in clinical populations of young people, there is evidence that early self-harm and suicidal behaviour predicts onset and chronicity of major mental illness [2]. Evidence from population studies may be inappropriately applied to suicide prevention in clinical populations if the relationship between particular risk factors, or processes, and suicidal behaviour differ between these two settings. For example, male gender is commonly cited as a significant risk factor for suicide in the population, but appears to be less associated with increased death due to suicide in clinical settings [3]. It has also been argued that the phenomenological differences between non-suicidal deliberate self-harm and suicide attempts that have been reported in population samples may not be as readily apparent in clinical populations [4]. In population samples, non-suicidal self-harm is associated with less lethality or severity, more repetition and a greater likelihood of cutting than overdose or other high-risk methods [5, 6]. Kapur et al. have argued that in clinical settings, episodes of non-suicidal self-harm are not so easy to distinguish from episodes of self-harm with suicidal intent, as they may be in non-clinical populations [4]. In clinical populations having multiple, interacting risk factors is the norm, rather than the exception, and understanding how those factors interact is complex [7].

Studies of complex processes have been aided by developments in machine learning (ML), which allows for the analysis of the high-dimensional data sets, where the number of potential predictors exceed the outcome of interest. Initial ML studies in suicide research have focused on identifying those at risk of suicidal behaviour in generalist settings such as emergency departments or military health services, rather than in mental health services [8, 9]. Though important, these findings may over-emphasise the clinical utility of such models in mental health services where the prevalence of suicidal ideation and behaviour and associated risk factors is higher, and identification of suicidality via assessment and screening is much more likely to be standard practice [10, 11].

Theories of suicidal behaviour, such as the Ideation-to-Action framework, have also emphasised the limited clinical utility of research that identifies predictors of suicidal behaviour relative to healthy controls, as these features will not necessarily distinguish those with suicidal ideation from those who engage in a suicidal act, which is the focus of clinical services [6]. These limitations are important in machine learning, because performance metrics such as accuracy are influenced by the ratio of those with the outcome to those without, that is the prevalence of the outcome in a population [12, 13]. Recent reviews of predictive modelling of future suicidal behaviour have emphasised the importance of understanding sample and setting when interpreting performance metrics [13] and have recommended developing predictive models specific to sub-populations where positive predictive value is likely to be high enough to meaningfully guide adoption of clinical interventions within services [14, 15].

The current study draws on the approach used by Iorfino et al., which applied a set of machine learning (ML) algorithms to routinely collected clinical information to develop predictive models of self-harm and suicidal behaviour in young people accessing mental health care services [16]. Although the use of routinely collected administrative data and/or clinical information in electronic health records (EHR) has been a focus of much machine learning health research, variables collected in EHRs can have limitations. For example, many EHRs may include diagnostic data, but not validated measures of symptoms or psychological constructs.

In the current study, a set of machine learning algorithms were applied to a comprehensive assessment battery containing validated self-report and clinician measures of social, clinical and psychological risk factors collected in a cohort of young people accessing community-based mental health services [17]. The set of algorithms was used to build four distinct ML models designed to address key questions facing clinical services delivering suicide prevention interventions. The study first aimed to establish performance of models predicting DSH or SA in the entire cohort, and whether performance differed by outcome. The second aim was to establish the performance of models predicting outcomes in key sub-groups, those with new-onset DSH or SA, and those who repeat DSH or SA. Finally, the study aimed to assess the relative importance of clinical and psychosocial factors predicting these outcomes.

## Methods

This study describes a longitudinal cohort study of 802 young people aged 12–25 years old accessing primary youth mental health care, known as the Transitions study [17]. The Transitions study was designed to assess a range of clinical, social and functional outcomes (including suicidal behaviour) of young people accessing youth-specific primary mental health services, known as ‘headspace’ clinics [17, 18]. Young people can self-refer, or be referred by clinicians, family members or school counsellors. Although the services provided by individual headspace clinics varies, the clinics included in this study contained a mix of primary mental health care and more specialised mental health services. Therefore, the presentations of young people attending these clinics ranges from non-specific and early-stage presentations to more discrete episodes of mental illness, including major mood disorder and psychosis [17].

The current study includes 802 young people who attended one of four clinics in Melbourne or Sydney, between January 2011 and August 2012. 509 young people completed a follow-up assessment at 12 months. Participants were invited to participate in the study by a trained research assistant (RA) after their initial clinical visit. Further details of the study methodology have been previously published [17]. The Strengthening the Reporting of Observational Studies in Epidemiology (STROBE) guidelines were used [19].

### Social and clinical assessment

The assessment battery included validated measures of clinical and psychosocial risk factors, which included both interviewer-led and self-report measures [17]. Measures included as part of the baseline assessment are detailed in Table 1 and are also detailed in the original study protocol [17]. Participants completed comprehensive assessments at two timepoints, baseline and 12-month follow-up. Assessment interviews were conducted by RAs, who were trained in use of study assessment measures and achieved an inter-rater reliability of at least 0.8 for interview-led measures. Demographic factors collected included age, gender, sexual orientation, ethnicity and country of participant’s birth and parent’s birth.

**Table 1:** Social and clinical assessment battery

Domain	Sub-domains	Measure	Interpretation
Demographics			
	Age	In years	
	Gender		Male, female, other
	Sexuality		Heterosexual or same sex attracted
Clinical symptoms (clinician-rated)			
Depression	Mood and cognitive symptoms	QIDS-8 [21]	Higher score denotes greater symptom severity
	Suicidal ideation	QIDS, item 12	Yes/no, where a score of 2 or more indicates yes
Sleep and energy	Initial insomnia	QIDS, item 1	
	Mid-nocturnal insomnia	QIDS, item 2	
	Early morning wakening	QIDS, item 3	
	Hypersomnia	QIDS, item 4	
	Fatigue	QIDS, item 14	
	Psychomotor slowing	QIDS, item 15	
	Psychomotor agitation	QIDS, item 16	
Mania	Mania symptoms	YMRS, 11 items [22]	Higher score indicates greater symptom severity in the last 48 h
Clinical stage	Stage 1A	Clinical staging model [23]	Non-specific symptoms
	Stage 1B		Attenuated syndrome
	Stage 2 +		First episode of discrete disorder or persistent, recurrent mental illness)
Clinical symptoms (self-report^a^)			
Rumination	Rumination	Rumination scale, 10 items [24]	Higher score indicates greater rumination
Psychological distress	Psychological symptoms	SPHERE-12, 6 items [25]	Higher score indicates greater symptom severity
	Somatic symptoms	SPHERE-12, 6 items	
Anxiety	Generalised anxiety symptoms	GAD, 7 items [26]	Higher score indicates greater symptom severity
	Frequency, severity and impact of anxiety symptoms	OASIS, 5 items [27]	Higher score indicates greater symptom severity
Disordered eating and associated behaviours	Preoccupation with weight or shape Purging Weight loss	SCOFF, 5 items [28]	Higher score indicates greater symptom severity
Substance use	Alcohol	WHO-ASSIST, Q2 [29]	High risk/ low risk High risk indicates at least monthly use in last 3 months
	Cannabis		
	Tobacco		
	Other illicit drugs (excluding above)	WHO-ASSIST, Q1	High risk/ low risk High risk indicates any history of use over the lifetime
Psychological and developmental factors (self-report^a^)			
Personality	Reward-responsiveness	BIS/BAS, 24-item scale [30]	Higher score indicates higher expression of trait
	Fun-seeking		
	Drive		
	Behavioural inhibition		
Childhood abuse	Physical abuse	CTQ, sub-scales [31]	Higher score indicates greater severity of abuse history
	Sexual abuse		
	Emotional abuse		
Parenting	Care	PBI [32] Paternal Maternal	Higher score indicates greater experience of sub-domain
	Over-protectiveness		
	Authoritarian		
Social factors (self-report^a^)			
Current employment or education	Education or employment, full-time or part-time	NEET [33]	Yes/no, where no indicates not in either employment or education
Social and occupational functioning	Level of functioning (clinician-rated)	SOFAS [34]	Higher score indicates higher functioning
Financial stress	Difficulty paying rent or utilities in last 6 months	ABS 2006 census [33]	Higher score indicates greater severity of stress
Recent adverse life events	Adverse life events	Brugha life stress scale, 11 items [35]	Higher score indicates greater severity of stress
Social support	Support from friends	Schuster Social support scale, 20 items [39]	Higher score indicates greater experience of sub-domain
	Support from family		
	Conflict with friends		
	Conflict with family		

Somatic symptoms: refers to symptoms that may occur as part of a somatisation process whereby psychological distress is experienced as physical symptoms, including musculoskeletal pain, fatigue, poor sleep and/or gastrointestinal symptoms (nausea, diarrhoea, constipation)

*ABS* Australian Bureau of Statistics, *BIS/BAS* behavioural inhibition/behavioural activation system, *CTQ* childhood trauma questionnaire, *GAD-7* generalised anxiety disorder scale, *NEET* Not in education or employment, *PB* parental bonding instrument, *QIDS* quick inventory of depressive symptoms, *SPHERE* somatic and psychological health report, *SOFAS* social and occupational functioning assessment scale, *OASIS* overall anxiety severity and impairment scale, *WHO-ASSIST* World Health Organisation Alcohol, Smoking and Substance Involvement Screening Test

^a^self-report unless otherwise indicated

### Self-harm and suicidal behaviour outcome measures

Questions assessing deliberate self-harm (DSH) and suicide attempts (SA) in the 12 months prior to each timepoint were interviewer-led and were previously adapted by Patton et al. from the Beck Suicide Inventory [20]. Further details of the assessment are included in Supplementary material Table 1. To ensure that participants who engaged in both deliberate self-harm and suicide attempts were not double counted, those who engaged in a suicide attempt were analysed in the suicide attempt group. History of DSH and/or suicide attempt was coded into three categories based on presence of a behaviour in the 12 months prior to each assessment; (1) those without self-harm, (2) a deliberate self-harm group (with DSH but had no suicide attempts); and (3) a suicide attempt group (those who reported having made a suicide attempt and may or may not have engaged in DSH also).

### Statistical analysis

Baseline social and clinical factors were analysed by outcome at 12 months, using chi-squared tests for categorical predictors and ANOVA for continuous predictors. The Bonferroni method was used to adjust for multiple testing. Post hoc tests were carried out using Scheffe’s test to determine where significant differences were. The baseline characteristics of the 293 participants who did not complete follow-up assessment were compared to the remaining sample, using t-tests for continuous variables and chi-squared tests for categorical variables (Supplementary table 3). Missing data from the 509 participants who did complete follow-up were inspected and found to be consistent with a missing-at-random pattern (Supplementary table 2). No variable was missing more than 10% of data. Missing data were imputed using the ‘multivariate imputation by chained equations’ (‘mice’) package in R [21]. Five imputations were run, using predictive mean matching for continuous variables and logistic regression for categorical variables, both used all available data. Imputed datasets were modelled separately, and coefficients, standard errors and test statistics were pooled. All analyses were performed in R (version 3.6.3) [22].

### Model building

**Fig. 1:**
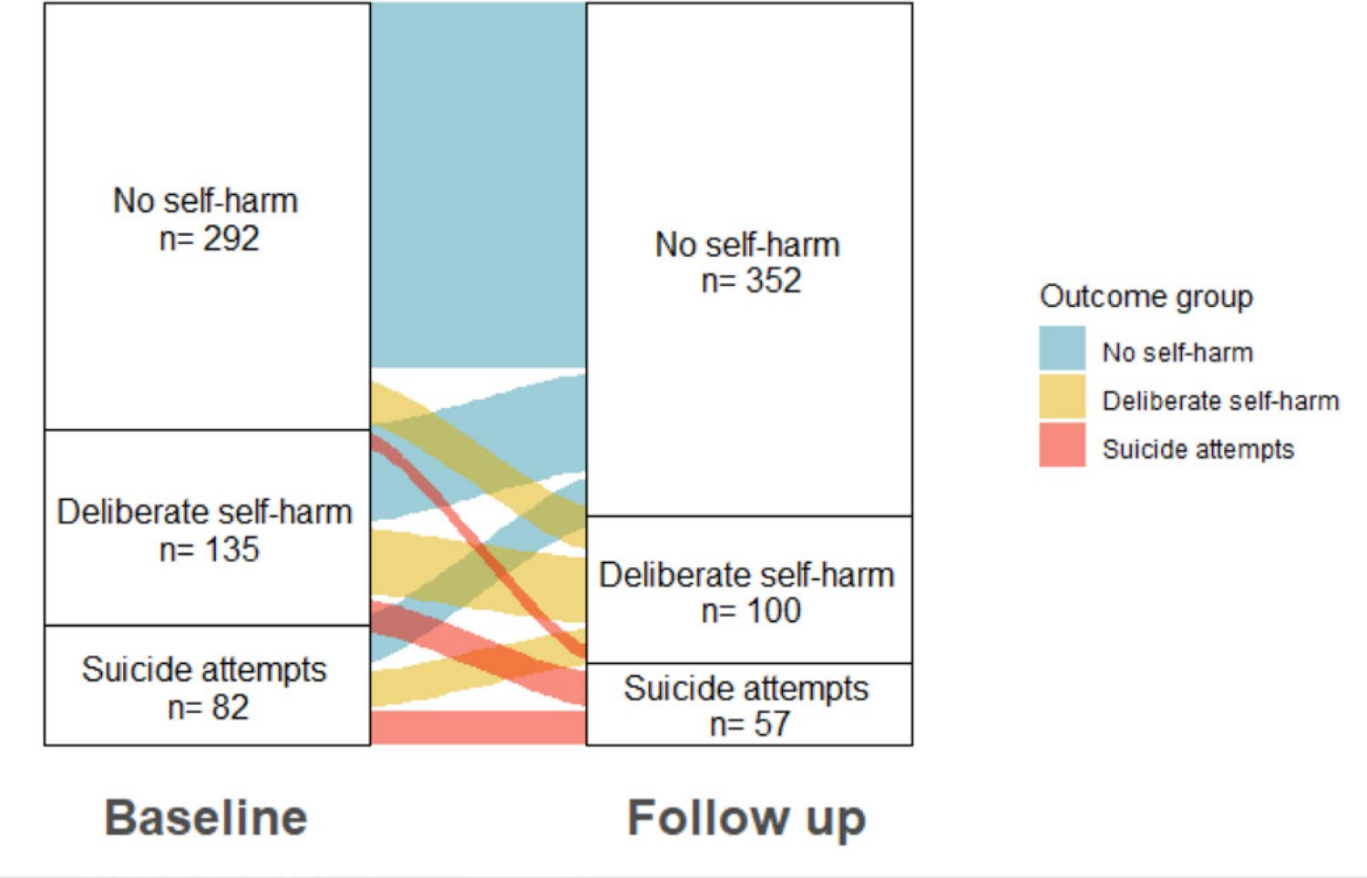
Machine learning model building. Model 1 predicts all participants who report DSH at follow-up (*n* = 100, yellow group) relative to those with no self-harm at follow-up (*n* = 352, blue group). Model 2 predicts participants who report SA at follow-up (*n* = 57, red group) relative to those with no self-harm at follow-up (*n* = 352) (blue group). Model 3 samples only those without self-harm at baseline (*n* = 292). Model 4 samples only those with either DSH or SA at baseline (*n* = 217)

We applied four different machine learning algorithms, as well as logistic regression, to build four different machine learning (ML) models representing different clinical samples to address our key questions (Fig. 1). The term ML model is used here to refer to a set of algorithms. Each algorithm was run for 100 trials and thus, the performance of each model represents the mean performance metrics over 500 trials (i.e. 5 algorithms × 100 trials).

*Model 1* compared the group of participants who reported DSH at follow-up (but no SA), to all participants who reported no self-harm during follow-up. *Model 2* compared all participants who reported SA during follow-up, to all who reported no self-harm during follow-up. Model 3 and 4 analysed predictors of specific sub-groups, those who repeated DSH or SA during care, or those who had new-onset DSH or SA during care. *Model 3* sampled only participants who reported no self-harm at baseline and compared those who reported new-onset DSH or SA at follow-up with those who had no self-harm at follow-up. *Model 4* sampled only participants who reported either DSH or SA at baseline and compared those who went on to repeat either behaviour during follow-up to those who reported no further DSH or SA.

### Machine learning algorithms and regression

Each ML model was built by applying multiple machine learning algorithms to the set of 50 potential predictors (Table 1). This approach is designed to improve predictive performance of a model, as well as the validity of variable selection out of a large number of potential predictors [12]. We followed the methods described in Demetriou et al. [23].The algorithms selected were Area Under the Curve Random Forests (AUCRF), Boruta, Least absolute shrinkage selection operator (LASSO) regression, elastic-net regression and logistic regression. In this study LASSO and elastic-net regression were forms of logistic regression given the binary outcome. Both AUCRF and Boruta are variants of the random forest (RF) algorithm. For each RF model we generated 1000 decision trees. For both RF (AUCRF and Boruta) and the penalised logistic regression techniques (LASSO and elastic-net regression), we used the internal fivefold cross-validation to identify the optimal lambda and alpha coefficient. Lambda controls the strength of penalization in LASSO and elastic-net regression and alpha controls the balance between L1- and L2-regularisation in elastic-net regression. Logistic regression, using a backwards stepwise approach, was included to allow for comparability between the other four algorithms and traditional statistical approach.

Another common problem faced in suicide research using machine learning is the issue of unbalanced datasets [12]. To account for unbalanced samples the synthetic minority over-sampling technique (SMOTE) algorithm was used [24], which allows for the number of patients in the minority class to be doubled by synthetic generation of cases. Borderline samples were identified via Gower distance and removed in order to increase the separation between sample classes [25]. Additionally, random under sampling of the majority class was used to balance with the minority class.

### Model validation and performance

Models were built and trained using 10 repeats of tenfold cross-validation, to address the risk of over-fitting the data as can occur in high-dimensional data sets [26, 27]. In each fold, samples were trained on 90% of data, and tested on 10% of data. The test set remained unaltered to assess the ML models’ performance on the real-world prevalence of deliberate self-harm and suicide attempts. Where the outcome variables were balanced, neither SMOTE nor random under sampling was implemented.

The performance of each algorithm within a model was compared using metrics including Area Under the Receiver-Operating Curve (AUROC), Area under the Precision–Recall Curve (AUPRC), sensitivity, specificity, brier score and positive predictive value. AUROC is a measure of the ability of a statistical model to discriminate between a binary outcome at all possible thresholds between 0 and 1. The ROC curve plots the true positive rate (also referred to as sensitivity or recall) against the false-positive rate (1 − specificity). An AUROC of 0.5, indicates a statistical model is no better at predicting an outcome than chance. An AUROC > 0.8 would generally be considered a strong performance, and 0.7–0.8 as good, 0.6–0.7 as sufficient and 0.5–0.6 as weak [28]. The area under the precision–recall curve is considered a more valid measure of performance of imbalanced datasets when detecting positive cases is important [29] The AUPRC plots the precision against recall, and hence the baseline for comparison should be the prevalence of the outcome in the sample. For example, if the prevalence of an outcome is 20%, then an AUPRC of 0.6 would be considered good, whereas if the prevalence is 60% an AUPRC of 0.6 would indicate the performance of the algorithm is no better than chance. Brier score measures discrimination and calibration, that is the magnitude of error in the probability estimates [30]. A brier score ranges from 0 (perfect accuracy) to 1 (perfect inaccuracy). Positive predictive value (PPV) was calculated for each algorithm to allow for an assessment of how clinically useful such an algorithm, and the ML model it informs, may be. PPV is influenced by the prevalence of an outcome in the sample and is dependent on a specific probability threshold, unlike AUROC and AUPRC.

### Variable selection

As each of the five algorithms produced 100 trials, we present summarised statistics for variable selection. For AUCRF and Boruta, a variable is deemed selected if it is present in the final iteration of the random forest trial algorithm. For the remaining regression-based techniques, a variable is deemed selected if the coefficient is not zero.

The frequency with which a variable was selected was summed across the 500 trials and ranked against other variables for each ML model. Final variable importance was determined based on whether a variable was selected in greater than 70% of trials per algorithm. Variables were also ranked by frequency of selection in descending order, with those ranked 1–10 indicating greatest importance.

## Results

Of the 802 participants who completed the baseline assessment, 509 participants (mean age 18.3 years, 69% females) completed the follow-up assessment at 12 months. Factors associated with the 37% (293/802) lost to follow-up were being of male gender, having lower baseline social and occupational functioning, being of earlier clinical stage and being more likely to report lifetime drug use (Supplementary table 3). Bivariate associations between baseline clinical and social variables and outcome group are presented in Supplementary Table 4.

### Prevalence and method of self-harm and suicide attempts at baseline

At baseline, 26.7% (214/802) reported engaging in deliberate self-harm but no suicide attempts and 15.8% (128/802) reported having made a suicide attempt (Table 2). All those who reported a suicide attempt had also engaged in an episode of deliberate self-harm. Therefore 42.3% (342/802) engaged in at least one episode of deliberate self-harm in the 12 months prior to baseline. Cutting or burning was the most common method of deliberate self-harm, reported by 70% (238/342) of those who self-harmed, while overdose or poisoning was the most common method of suicide attempt, reported by 50% (64/128) of those who reported a suicide attempt. Engaging in multiple methods of self-harm or suicidal behaviour was also common, with more than 38% (130/342) reporting engagement in two or more methods.

**Table 2:** Prevalence of suicidal behaviour at baseline and follow-up

Baseline	Follow-up				
	No self-harm	Deliberate self-harm	Suicide attempt	No follow-up	Total
No self-harm, *n* (%)	251 (54.6)	30 (6.5)	11 (2.4)	168 (36.5)	460 (100)
Deliberate self-harm, *n* (%)	67 (31.3)	45 (21.0)	23 (10.7)	79 (36.9)	214 (100)
Suicide attempt, *n* (%)	34 (26.6)	25 (19.5)	23 (18.0)	46 (35.9)	128 (100)
Total	352	100	57	293	802

### Prevalence and rates of repetition of self-harm and suicide attempts at follow-up

Of the 509 participants assessed at follow-up 11.2% (57 of 509) reported having made a suicide attempt and engaged in deliberate self-harm and 19.6% (100 of 509) reported an episode of deliberate self-harm without suicide attempt in the 12-month follow-up period. All those who made a suicide attempt also reported an episode of DSH. The proportion of young people with DSH or SA at baseline, who repeated DSH or SA in 12 months of follow-up was high (53.5%,116/217). Of those who completed follow-up and had no history of self-harm or suicide attempts at baseline, 14% (41 of 292) reported new-onset self-harm or suicidal behaviour (DSH without SA 10.3%, SA 3.8%) in the follow-up period.

**Fig. 2:**
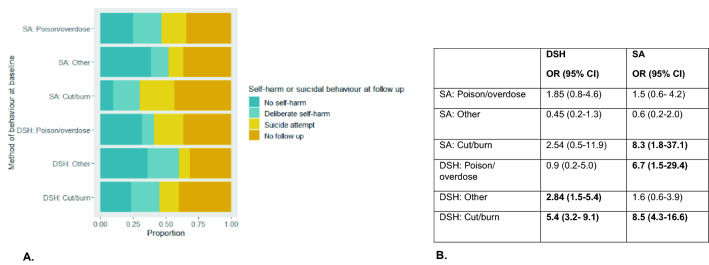
Method of behaviour at baseline and likelihood of further DSH or SA. Samples only those young people who reported DSH or SA at baseline (*n* = 342) and includes those lost to follow-up. **A** presents the proportion of young people in each outcome group by method of DSH or SA at baseline, and **B** presents the odds ratio of either outcome, DSH or SA, according to baseline behaviour and method. Reference group for odds ratio calculations was those who completed follow-up and reported no further self-harm

Risk of DSH or SA during follow-up varied by type of baseline self-harm behaviour (i.e. intent and method used) (Fig. 2A). Young people who reported having made a suicide attempt at baseline assessment were twice as likely to report having made a suicide attempt during follow-up than those who had engaged in DSH only at baseline (OR 15.4, CI 6.9–34, vs. OR 7.8, CI 3.6–16.9); however, it should be noted that both ORs were associated with a wide confidence interval, which overlapped. DSH via cutting or burning at baseline was associated with the greatest odds of either DSH or SA during follow-up (see Fig. 2B). Suicide attempt at baseline by cutting or burning and DSH by poison or overdose were both also associated with an increased OR of suicide attempt during follow-up.

### Machine learning algorithms and model selection

The mean and standard deviation for performance metrics of each algorithm, AUCRF, Boruta, Elastic-net regression, LASSO and logistic regression, are described in Table 3. The performance of each algorithm on the training dataset is available in Supplementary material (Supplementary Fig. 1A–D). The elastic-net regression algorithm was associated with a slightly better mean AUROC in each ML model, aside from Model 3 where it was ranked behind LASSO and Boruta. Logistic regression, which was included for comparison, was ranked 5th in terms of performance metrics in each ML model, aside from Model 1, where Boruta was ranked last and logit performance was on par with the AUCRF. Aside from logistic regression, which was associated with reduced performance, other algorithms had similar performance.

**Table 3:** Performance metrics of each algorithm by model

ML model	Sample	Algorithm	AUROC, mean (SD)	AUPRC, mean (SD)	Brier score, mean (SD)	Sensitivity, mean (SD)	Specificity, mean (SD)	PPV, mean (SD)
Model 1 (*n* = 452)	DSH at follow-up (*n* = 100) vs. No self-harm at follow-up (*n* = 352) Prevalence of outcome 22%	AUCRF	0.71 (0.10)	0.39 (0.10)	0.20 (0.02)	0.57 (0.16)	0.75 (0.08)	0.40 (0.11)
		Boruta	0.68 (0.11)	0.37 (0.11)	0.21 (0.03)	0.56 (0.16)	0.74 (0.08)	0.38 (0.10)
		Elastic-net	0.74 (0.09)	0.42 (0.12)	0.20 (0.03)	0.62 (0.16)	0.74 (0.08)	0.41 (0.09)
		LASSO	0.74 (0.09)	0.42 (0.11)	0.20 (0.03)	0.62 (0.15)	0.75 (0.07)	0.42 (0.09)
		Logit	0.71 (0.09)	0.39 (0.11)	0.24 (0.05)	0.61 (0.15)	0.70 (0.08)	0.37 (0.07)
Model 2 (*n* = 409)	SA at follow-up (*n* = 57) vs. No self-harm at follow-up (*n* = 352) Prevalence of outcome 13.9%	AUCRF	0.79 (0.09)	0.32 (0.12)	0.17 (0.03)	0.71 (0.18)	0.75 (0.07)	0.32 (0.09)
		Boruta	0.80 (0.10)	0.34 (0.13)	0.17 (0.03)	0.69 (0.19)	0.76 (0.08)	0.33 (0.11)
		Elastic-net	0.82 (0.09)	0.36 (0.11)	0.17 (0.04)	0.71 (0.20)	0.78 (0.07)	0.35 (0.11)
		LASSO	0.82 (0.09)	0.35 (0.11)	0.18 (0.04)	0.70 (0.20)	0.78 (0.07)	0.35 (0.10)
		Logit	0.68 (0.11)	0.10 (0.09)	0.29 (0.08)	0.61 (0.18)	0.72 (0.09)	0.27 (0.09)
Sub-group analyses								
Model 3 (*n* = 292)	New-onset DSH or SA (*n* = 41) vs. No history of self-harm at BL or FU (*n* = 251) Prevalence of outcome 14%	AUCRF	0.57 (0.14)	0.17 (0.06)	0.23 (0.04)	0.39 (0.25)	0.69 (0.10)	0.17 (0.10)
		Boruta	0.58 (0.15)	0.17 (0.06)	0.22 (0.03)	0.41 (0.25)	0.70 (0.10)	0.18 (0.10)
		Elastic-net	0.57 (0.15)	0.18 (0.08)	0.26 (0.06)	0.43 (0.26)	0.67 (0.12)	0.18 (0.12)
		LASSO	0.58 (0.14)	0.18 (0.08)	0.26 (0.05)	0.43 (0.25)	0.67 (0.12)	0.18 (0.11)
		Logit	0.51 (0.14)	0.12 (0.07)	0.41 (0.10)	0.45 (0.24)	0.58 (0.11)	0.15 (0.08)
Model 4 (*n* = 217)	Repeat DSH or SA (*n* = 116) vs. No repeat DSH or SA (*n* = 101) Prevalence of outcome 53.4%	AUCRF	0.61 (0.11)	0.59 (0.09)	0.26 (0.04)	0.50 (0.14)	0.66 (0.15)	0.64 (0.13)
		Boruta	0.62 (0.11)	0.59 (0.09)	0.27 (0.04)	0.50 (0.14)	0.67 (0.14)	0.64 (0.12)
		Elastic-net	0.64 (0.11)	0.60 (0.08)	0.25 (0.03)	0.47 (0.15)	0.71 (0.15)	0.66 (0.12)
		LASSO	0.62 (0.12)	0.59 (0.09)	0.25 (0.03)	0.45 (0.17)	0.71 (0.16)	0.38 (0.19)
		Logit	0.55 (0.12)	0.54 (0.08)	0.35 (0.07)	0.46 (0.14)	0.61 (0.16)	0.58 (0.12)

Each algorithm was run over 100 trials, thus results of 500 trials were used to calculate mean and standard deviation of each performance metric. In the ML models with samples that were unbalanced in terms of outcome groups (Models 1–3) random under sampling of controls and synthetic generation of cases were used to adjust for this. ML Model 4 which compared participants with repeat DSH/SA versus those without repeat behaviour was balanced by outcome so these techniques were not required

*AUCRF* area under the curve random forest, *AUROC* area under the receiver-operator curve, *AUPRC* area under the precision–recall curve, *BL* baseline, *DSH* deliberate self-harm, *FU* follow-up, *ML* machine learning, PPV positive predictive value, *SA* suicide attempt, *SD* standard deviation

### Model performance

The mean performance of models predicting either DSH (Model 1) or SA (Model 2) were both strong. The ML model predicting SA was associated with greater mean performance metrics than the model predicting DSH (SA: AUC: 0.82 sensitivity: 0.72, specificity: 0.78, DSH: AUC: 0.72 sensitivity: 0.62, specificity: 0.73). The mean positive predictive value (PPV) of the ML model predicting SA was approximately twice that of the prevalence (SA prevalence 14%, PPV: 0.32), as was the ML model predicting DSH (prevalence 22%, PPV: 0.4).

Model 3 which tested the ability of the algorithms to predict new-onset DSH or SA during follow-up amongst participants who reported no history of DSH or SA at baseline performed poorly (AUROC 0.55, AUPRC: 0.16, sensitivity: 0.26, specificity: 0.76). Both AUROC and AUPRC indicated that Model 3 is little better than chance at predicting new-onset DSH or SA. Model 4 which tested the ability of the algorithms to predict repetition of DSH or SA among those who reported either behaviour at baseline, performed slightly better (AUROC 0.63, sensitivity: 0.53, specificity: 0.67). The mean positive predictive values of Models 3 and 4 were only slightly greater than prevalence (Model 3 prevalence 14%, PPV: 0.17, Model 4 prevalence 53%, PPV: 0.63).

### Variable selection

**Fig. 3:**
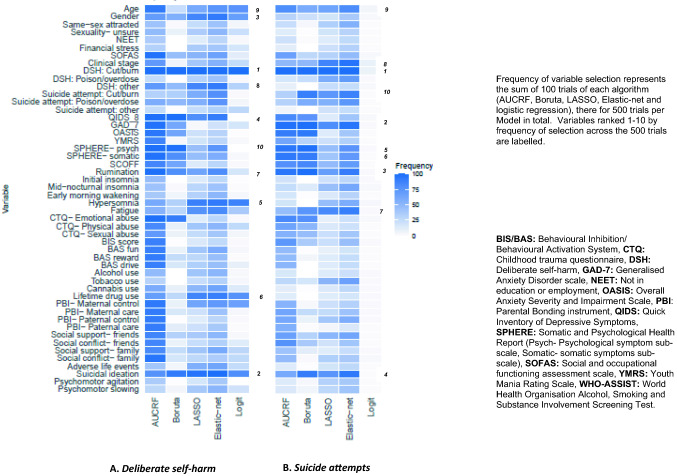
Heatmap displaying frequency of variable selection by each algorithm in ML Model 1 and Model 2. Frequency of variable selection represents the sum of 100 trials of each algorithm (AUCRF, Boruta, LASSO, Elastic-net and logistic regression), there for 500 trials per Model in total. Variables ranked 1–10 by frequency of selection across the 500 trials are labelled. **A** presents variable selection in Model 1 (deliberate self-harm) and **B** presents variable selection in Model 2 (suicide attempts)

Models 1 and 2 were used for variable selection as the performance metrics of these models indicated they were accurate enough to be used for variable selection. Models 3 and 4 were no better at predicting outcomes than chance, thus using these models for variable selection was not appropriate. Figure 3 presents a heatmap of the frequency of variable selection by each algorithm in Model 1 (Fig. 3A) and 2 (Fig. 3B). History of deliberate self-harm via cutting at baseline was the most frequently selected predictor in both models, being selected in all 500 trials of algorithms predicting DSH at follow-up and 408 trials of algorithms predicting SA at follow-up. While half of the variables ranked 1–10 by frequency of selection across algorithm trials were common to models of both outcomes (DSH via cutting, suicidal ideation, general psychological distress, rumination and younger age), there was also evidence for a distinct pattern of variable selection between the models predicting DSH or SA. Symptoms of generalised anxiety, fatigue, somatic symptoms, a history of suicide attempt by cutting, more advanced clinical stage (> stage 1B), and eating disorder symptoms, were variables important to the prediction of SA (i.e. Model 2, Fig. 3B) but not DSH (i.e. Model 1, Fig. 3A). Variables that were ranked within 1–10 in models of DSH but not SA were an absence of hypersomnia, female gender, depressive symptoms (QIDS-8), a history of recreational drug use, and deliberate self-harm by engaging in high-risk behaviours.

**Fig. 4:**
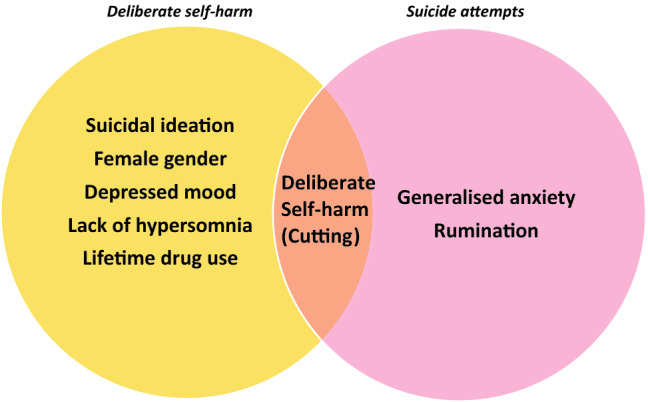
Variables important to the prediction of DSH and/or SA. Important variables were those that were selected in at least 70% of algorithm trials

Figure 4 presents variables selected in at least 70% of algorithm trials. Those selected in algorithms predicting DSH were suicidal ideation (446/ 500), female gender (374/ 500), depressive symptoms (QIDS-8) (371/500), absence of hypersomnia (365/500), and lifetime drug use (350/500). After DSH by cutting, only two other variables were selected in at least 70% of algorithm trials predicting SA, generalised anxiety symptoms (GAD-7) (377/500) and ruminative thinking (358/500).

## Discussion

Machine learning models predicting deliberate self-harm and suicide attempts differed in terms of their ability to predict future events, as well as in the variables selected to do so. Both models predicting either DSH or SA performed well, though the model predicting SA was superior with greater mean AUROC, as well as greater mean sensitivity and mean specificity. While a number of variables were frequently selected in both, there was also evidence that some factors were important to either DSH or SA models but not both (Fig. 4). Our findings suggest risk factors within clinical populations of young people are different to those of population samples of young people. For example, our findings suggest that in clinical samples, method of self-harm or suicidal behaviour may be as important as whether the behaviour is associated with suicidal intent in determining future risk of suicide attempt. For example, cutting, with or without suicidal intent was more important in predicting future suicide attempt than other methods where suicidal intent was present. Similarly, deliberate self-harm (without suicidal intent) via poisoning was more frequently selected by algorithms predicting future suicide attempts, but not deliberate self-harm. In population samples, overdose and poisoning are usually more strongly associated with suicide attempts than non-suicidal self-harm [6], and our findings suggest that in clinical samples an overdose should be considered a greater risk of future suicidal behaviour regardless of whether suicidal intent is expressed.

Aside from age and a history of self-harm or suicide attempts the highest-ranking variables in each model were almost entirely validated symptom scales of common mental disorders, rather than other social or demographic factors. Although social factors are important risk factors at a population level, our findings suggest that common symptoms of mental disorder play a pivotal role in the expression of self-harm and suicidal behaviour in clinical settings. Rather than indicate that social factors are not important contributors to suicide risk it appears that such risk factors were similarly prevalent in young people in clinical settings with or without self-harm or suicidal behaviour. There was also evidence for a distinct pattern of predictors between the two outcomes. Gender was an important variable in the model of DSH but was not frequently selected in the prediction of SA, adding further evidence to the more limited effect of gender on suicidal behaviour in clinical services [31, 32]. More advanced clinical stage (> stage 1B), somatic symptoms, generalised anxiety and symptoms of disordered eating and disturbance of body image ranked highly in the prediction of SA but not DSH. These symptom clusters are often associated with more severe disorders in young people that tend to follow a more chronic or relapsing course. For example, fatigue and somatic symptoms are associated with more severe variants of depression, including bipolar depression and treatment resistant depression [33]. Co-morbid symptoms of generalised anxiety or disordered eating may increase treatment resistance [33].

Our findings of the importance of clinical symptoms to future self-harm or suicidal behaviour suggest that leading theories of suicidal behaviour may require adaption to clinical settings. Theorists have suggested that mental disorders contribute to suicidal behaviour via their effect on suicidal ideation or in certain conditions by increasing agitation, but not beyond that [34]. In the context of clinical services, it must be considered that a diagnosis of a major mental illness such as a moderate to severe major depressive episode, can account for many features of the leading ideation-to-action theories, including the experience of hopelessness, worthlessness, withdrawal from relationships and perceived isolation. The experience of mental illness, which is often episodic and relapsing in course, may also increase the likelihood of experiencing prolonged or recurrent symptoms and, thus, result in greater degree of social and functional impairment and have a greater impact on an individual’s sense of belonging and development of intimate relationships. Further research into the impact of symptom improvement on future suicidal behaviour in cohorts of young people accessing clinical care is needed.

Our second aim was to understand how predictive modelling performed when predicting new-onset or repeat DSH or SA. Models of new-onset or repeat DSH or SA were developed by sampling sub-groups of participants to understand how the models performed when baseline DSH or SA is known, which is often the case in clinical services. Both models performed poorly and were only slightly better at predicting outcome than chance. These findings re-affirm the importance of previous self-harm or suicidal behaviour in determining risk of future behaviour and suggest that performance of predictive modelling on heterogenous samples where history of suicidal behaviour is not controlled for may be inflated. Despite this, the prevalence of repeat DSH or SA (53.4%) in those who reported either at baseline was high enough to justify a set of enhanced clinical interventions for this group. On the other hand, the incidence of new DSH or SA, in those without either behaviour at baseline was similar to the incidence in the age-matched population [35]. Understanding what type of clinical suicide prevention interventions should be delivered to this sub-group remains a challenge. More longitudinal data on outcomes in this group may help clarify how suicide prevention should be prioritised relative to other clinical priorities. As highlighted by Bossarte et al. [36] future ML studies may move away from a focus on prediction of future suicidal behaviours, and instead focus on predicting response to treatment.

There were a number of limitations in this study. Although analysing high-dimensional data sets without over-fitting is considered a distinct advantage of machine learning, the smaller sample size of the sub-group analyses remains a limitation. A number of techniques were used to reduce the risk of over-fitting (cross-validation, test–train splitting, SMOTE). However, dimensionality reduction prior to predictive modelling, for example through principal component analysis, is another approach recommended to improve performance metrics and validity, and reduce any effect of collinearity [37]. The use of additional approaches to dealing with imbalanced data, such as weighting the algorithm loss function to penalise false-negative results more than false-positive results may have increased the robustness of the analysis [38].

As a rule, variable selection should be interpreted with caution in machine learning studies as ML has a limited ability to examine associations between predictors. In the current study some validated measures, were broken down into key symptom clusters and included as separate variables. This was decided a priori to examine specific symptom clusters that have been shown to have an association with suicidal behaviour independent of the disorder measured by the parent scale [39, 40]. However, this may have increased the interaction between variables, and thus reduced the validity of the variable selection process.

It should also be noted the current study focus on a community-based mental health service for young people (12–25 years) and collected history of DSH or suicide attempt via clinical interview. Loss to follow-up was also greater in those at an earlier clinical stage. This may limit the generalisability of the findings to other clinical mental health settings, such as emergency departments, or cohorts of different ages, or in samples with suicidal behaviour that has been confirmed by assessment during a hospital presentation.

## Conclusion

Modelling of future self-harm and suicidal behaviour should be specific to particular clinical settings and clinical questions. This is crucial as the performance of predictive models is related not only to the prevalence of the outcome of interest in the sample but also on whether a service or clinician has prior knowledge of the individual’s history of deliberate self-harm or suicide attempts. Predictors of DSH or SA in clinical populations of young people differ from those of population samples. Method of DSH or SA used may be as important to prediction of future behaviours as the expression of suicidal intent. While a history of DSH via cutting was the most important variable in predicting both outcomes, other highly ranked predictors represented a greater experience of clinical symptoms. Given the significant role clinical symptoms play in predicting deliberate self-harm or suicide attempts in young people accessing clinical services, the key suicide prevention in this cohort should be the delivery of high-quality clinical care that aims for recovery, of which amelioration of symptoms is one component.

## Supplementary Information

Below is the link to the electronic supplementary material.

Supplementary file1 (DOCX 115 KB)

## Data Availability

The Transitions cohort data is not open access but may be made available upon request to the original study authors and subject to ethical and organisational review.
