# Supplementary material for: Predictive modelling of deliberate self-harm and suicide attempts in young people accessing primary care: a machine learning analysis of a longitudinal study
Source: Soc Psychiatry Psychiatr Epidemiol. 2023 Feb 28;58(6):893–905. doi: 10.1007/s00127-022-02415-7 (PMC10241686; doi:10.1007/s00127-022-02415-7)

**Supplementary Table 1.** Assessment of self-harm and suicidal behaviour

|  | **Interviewer questions** |
| --- | --- |
| **Deliberate self-harm** | In the past 12 months have you deliberately hurt yourself or done anything that you knew might have harmed you? |
| ***Method*** | If so, how?^a^ |
| **Suicide attempt** | ‘In the past 12 months, did you do something to try and kill yourself?’. |
| ***Method*** | If so, how?^a^ |

^a^ Answers were recorded verbatim by interviewer and later coded by two researchers independently of each other, into the following categories, 1) cutting or burning, 2) overdose or poisoning, and 3) all other behaviours.

**Supplementary Table 2.** Degree of missingness in baseline variables

| Variable | Count (%) n=509 | % |
| --- | --- | --- |
| Age | 0 | 3.7 |
| Gender | 0 | 3.7 |
| Educational level | 19 | 3.7 |
| NEET status | 19 | 3.7 |
| SOFAS | 15 | 2.9 |
| Financial stress | 19 | 3.7 |
| Family history of suicide | 26 | 5.1 |
| Adverse life events scale | 23 | 4.5 |
| *Suicidal ideation and behaviour at baseline* | |  |
| Suicidal ideation | 1 | 0.2 |
| Deliberate self-harm | 1 | 0.2 |
| Suicide attempt | 1 | 0.2 |
| *Presenting symptoms* | |  |
| QIDS | 1 | 0.2 |
| YMRS | 2 | 0.4 |
| GAD-7 | 28 | 5.5 |
| SPHERE scale | 28 | 5.5 |
| SCOFF | 28 | 5.5 |
| OASIS | 27 | 5.3 |
| Rumination scale | 22 | 4.3 |
| Clinical stage | 9 | 1.8 |
| Alcohol current use | 1 | 0.2 |
| Cannabis current use | 1 | 0.2 |
| Tobacco current use | 1 | 0.2 |
| Lifetime other drug use | 1 | 0.2 |
| *Developmental factors* | |  |
| Rumination scale | 22 | 4.3 |
| Childhood trauma questionnaire | 27 | 5.3 |
| BIS/BAS scale | 22 | 4.3 |
| Parental bonding index: maternal | 26 | 5.1 |
| Parental bonding index: paternal | 41 | 8.1 |
| Social support scale | 25 | 4.9 |

**Supplementary Table 3.** Baseline characteristics of participants who were lost to follow-up relative to those who completed follow-up

|  | | | Lost to follow-up | Completed follow-up | test | p- value^†^ |
| --- | --- | --- | --- | --- | --- | --- |
| *n (%)* | | | 293 (36.5) | 509 (63.5) |  |  |
| *Social and demographic* | | |  |  |  |  |
| Age, in years, mean (SD) | | | 18.2 (3.2) | 18.3 (3.2) | t | 0.658 |
| Gender, n (%) *Female* |  | | 178 (60.8) | 351 (69.0) | X^2^ | 0.022 |
| *Suicidal ideation and behaviour at baseline* | | | | |  |  |
| Suicidal ideation, n (%) | | | 34 (11.6) | 82 (16.1) | X^2^ | 0.1 |
| Deliberate self-harm, n (%) | | | 79 (27.1) | 135 (26.5) | X^2^ | 0.890 |
| Suicide attempts, n (%) | | | 46 (15.8) | 82 (16.1) | X^2^ | 0.879 |
| *Presenting symptoms* | | |  | |  |  |
| QIDS- 16 item score, mean (SD) | | | 10.45 (5.1) | 10.15 (5.5) | t | 0.447 |
| Manic symptoms, mean (SD) | | | 4.45 (4.9) | 3.65 (4.7) | t | 0.022 |
| Generalised anxiety symptoms, mean (SD) | | | 10.04 (5.9) | 9.75 (6.0) | t | 0.516 |
| SPHERE psych score, mean (SD) | | | 5.64 (3.8) | 5.55 (3.8) | t | 0.736 |
| SPHERE somatic score, mean (SD) | | | 5.85 (3.7) | 5.35 (3.5) | t | 0.066 |
| Eating disorder symptoms, mean (SD) | | | 1.14 (1.3) | 1.15 (1.3) | t | 0.982 |
| OASIS, mean (SD) | | | 7.72 (5.5) | 7.45 (5.0) | t | 0.475 |
| Rumination thinking, mean (SD) | | | 28.84 (7.6) | 28.80 (6.9) | t | 0.946 |
| *Clinical staging and functioning* | | |  |  |  |  |
| Clinical stage (%) | | 1A | 111 (37.9) | 176 (34.6) | X^2^ | 0.002 |
|  | | 1B | 159 (54.3) | 253 (49.7) |  |  |
|  | | 2+ | 19 (6.5) | 77 (15.1) |  |  |
| SOFAS score, mean (SD) | | | 63.73 (11.3) | 66.21 (11.7) | t | 0.004 |
| *Substance use* | | |  |  |  |  |
| Alcohol current high (%) | | | 97 (33.1) | 190 (37.3) | X^2^ | 0.34 |
| Tobacco current high (%) | | | 24 (8.2) | 63 (12.4) | X^2^ | 0.001 |
| Cannabis current high (%) | | | 78 (26.6) | 125 (24.6) | X^2^ | 0.462 |
| Other drug lifetime use (%) | | | 119 (40.6) | 165 (32.4) | X^2^ | 0.022 |

^†^ p-value significance set at 0.003, adjusted using Bonferroni method for multiple testing

**Supplementary Table 4.** Baseline social and clinical variables by outcome group at follow-up

|  | | | Total | No self-harm (a) | DSH (b) | Suicide attempt (c) | Test | p- value^†^ | Post-hoc tests |
| --- | --- | --- | --- | --- | --- | --- | --- | --- | --- |
| *n (%)* | | | 509 | 352 (69) | 100 (19.6) | 57 (11.2) |  |  |  |
| ***Social and demographic*** | | |  |  |  |  |  |  |  |
| Age, in years, mean (SD) | | | 18.4 (3.26) | 18.53 (3.31) | 17.91 (3.02) | 17.56 (2.85) | *F* | 0.042 |  |
| Gender, n (%) *Female* |  | | 351 (70.0) | 221 (62.8) | 83 (83) | 47 (82) | X^2^ | <0.001 | ***c vs. a, ***b vs. a |
| ***Presenting symptoms*** | | |  |  |  |  |  |  |  |
| Suicidal ideation, n (%) | | | 76 (14.9) | 32 (9.1) | 28 (28.0) | 22 (38.6) | X^2^ | <0.001 | ****c vs. a,*  ****b vs. a,*  **c vs. b* |
| Initial insomnia, n (%) | | | 223 (43.8) | 167 (47.4) | 56 (56.0) | 37 (64.9) | X^2^ | 0.027 |  |
| Mid-nocturnal insomnia, n (%) | | | 82 (16.1) | 60 (17.0) | 22 (22.0) | 21 (36.8) | X^2^ | 0.002 |  |
| Early morning wakening, n (%) | | | 63 (12.4) | 43 (12.2) | 20 (20.0) | 14 (24.6) | X^2^ | 0.017 |  |
| Hypersomnia, n (%) | | | 83 (16.3) | 74 (21.0) | 9 (9.0) | 8 (14.0) | X^2^ | 0.016 |  |
| Fatigue, n (%) | | | 54 (10.6) | 36 (10.5) | 18 (18) | 18 (32.1) | X^2^ | <0.001 | ****c vs. a, ***b vs. a,*  ***c vs. b* |
| Psychomotor slowing, n (%) | | | 34 (6.8) | 23 (6.5) | 3 (3.0) | 8 (14.0) | X^2^ | 0.028 |  |
| Psychomotor agitation, n (%) | | | 52 (10.2) | 31 (8.8) | 10 (10.0) | 11 (19.3) | X^2^ | 0.053 |  |
| QIDS- 8 item score, mean (SD) | | | 10.1 (5.5) | 9.13 (5.19) | 12.08 (5.33) | 13.09 (5.97) | *F* | <0.001 | ****b vs. a*  ****c vs. a* |
| Manic symptoms, mean (SD) | | | 3.6 (4.7) | 3.39 (4.29) | 3.71 (5.20) | 5.00 (5.73) | *F* | 0.053 |  |
| Generalised anxiety symptoms, mean (SD) | | | 9.7 (6.0) | 8.76 (5.73) | 11.02 (5.93) | 13.54 (6.00) | *F* | <0.001 | ****c vs. a, **b vs. a, *c vs. b* |
| SPHERE psych score, mean (SD) | | | 5.5 (3.8) | 4.82 (3.69) | 6.68 (3.46) | 7.82 (3.60) | *F* | <0.001 | ****b vs. a, *** c vs. a* |
| SPHERE somatic score, mean (SD) | | | 5.3 (3.5) | 4.82 (3.49) | 6.10 (3.28) | 7.12 (3.22) | *F* | <0.001 | ***b vs. a, *** c vs. a* |
| Eating disorder symptoms, mean (SD) | | | 1.1 (1.2) | 0.97 (1.17) | 1.40 (1.34) | 1.67 (1.27) | *F* | <0.001 | **** c vs. a, ** b vs. a* |
| OASIS, mean (SD) | | | 7.4 (5.0) | 6.65 (4.89) | 8.70 (4.87) | 9.93 (4.84) | *F* | <0.001 | **** c vs. a, ** b vs. a* |
| Rumination thinking, mean (SD) | | | 28.8 (6.9) | 27.62 (6.95) | 30.60 (5.72) | 32.77 (5.99) | *F* | <0.001 | **** c vs. a, *** b vs. a* |
| ***Clinical staging and functioning*** | | |  |  |  |  |  |  |  |
| Clinical stage (%) | | 1A | 176 (34.6) | 141 (40.1) | 28 (28) | 7 (12.3) | X^2^ | <0.001 | ****c vs. a, b vs. a,*  **c vs. b* |
|  | | 1B | 253 (49.7) | 158 (44.9) | 56 (56) | 39 (68.4) |  |  | ****c vs. a, ***b vs. a* |
|  | | 2+ | 77 (15.1) | 50 (14.2) | 16 (16) | 11 (19.3) |  |  | ****c vs. a, **b vs. a* |
| SOFAS score, mean (SD) | | | 66.2 (11.7) | 67.41 (11.71) | 64.12 (11.64) | 62.42 (10.79) | *F* | 0.002 | **c vs. a,*  **b vs. a* |
| ***Substance use*** | | |  |  |  |  |  |  |  |
| Alcohol current high (%) | | | 190 (37.3) | 136 (38.6) | 32 (32.0) | 22 (38.6) | X^2^ | 0.167 |  |
| Tobacco current high (%) | | | 63 (12.3) | 38 (10.8) | 13 (13.0) | 12 (21.0) | X^2^ | 0.033 |  |
| Cannabis current high (%) | | | 125 (24.6) | 75 (21.3) | 31 (31.0) | 19 (33.0) | X^2^ | 0.052 |  |
| Other drug lifetime use (%) | | | 146 (28.7) | 103 (29.3) | 43 (43.0) | 19 (33.0) | X^2^ | 0.034 |  |

^†^ p-value significance set at 0.003, adjusted using Bonferroni method for multiple testing. Post-hoc pairwise test- Scheffe’s test, *p=0.01 **p= 0.001, *** p<0.0001, or fisher’s exact test for chi-squared tests.

**Supplementary Figure 1A.** Performance of test and train datasets for Model 1 (predicting DSH only)


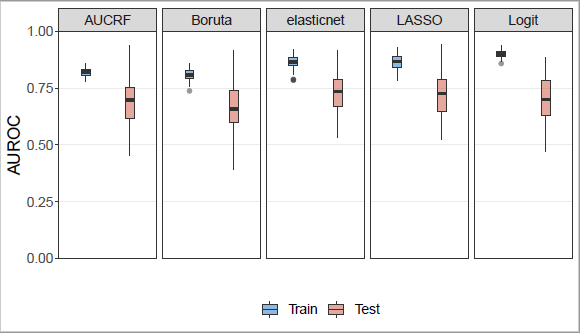


**Supplementary Figure 1B.** Performance of test and train datasets for Model 2 (predicting SA)


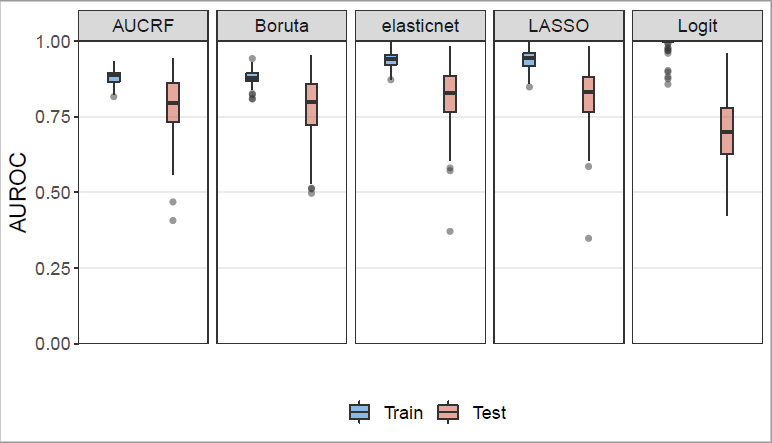


**Supplementary Figure 1C.** Performance of test and train datasets for Model 3 (New onset DSH or SA)


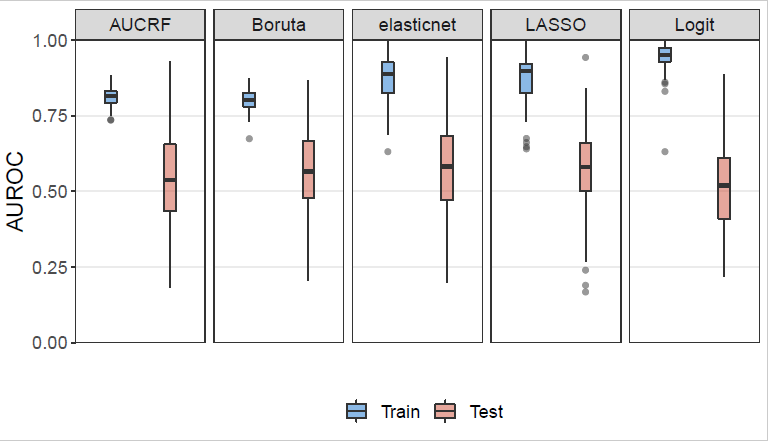


**Supplementary Figure 1D**. Performance of test and train datasets for Model 4 (Repeat DSH or SA)


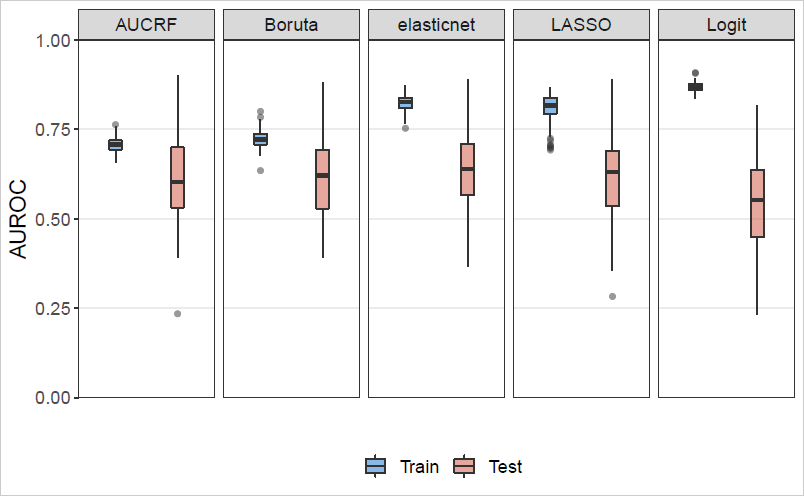

Supplement: Supplementary file 1 — Supplementary file1 (DOCX 115 KB) [file 127_2022_2415_MOESM1_ESM.docx]
